# Comparison between erbium-doped yttrium aluminum garnet laser therapy and sling procedures in the treatment of stress and mixed urinary incontinence

**DOI:** 10.1007/s00345-018-2445-x

**Published:** 2018-08-16

**Authors:** Nobuo Okui

**Affiliations:** Uro-Gyn.Net Health Care Cooperation, Dr. Okui’s Urogynecology and Urology Clinic, Kanagawa, Japan

**Keywords:** Non-ablative erbium yttrium aluminum garnet laser, Tension-free vaginal tape, Transobturator tape, One-hour pad test, International Consultation on Incontinence Questionnaire Short Form, Overactive bladder symptom score

## Abstract

**Purpose:**

Stress urinary incontinence (SUI) and mixed urinary incontinence (MUI) lead to poor quality of life. In Japan, urinary incontinence is treated with tension-free vaginal tape (TVT) or transobturator tape (TOT) sling procedures, which involves inserting a synthetic material; however, problems arise with artificial mesh in some instances, requiring new treatment methods. Hence, laser therapy, whereby an erbium-doped yttrium aluminum garnet laser is directed into the vagina and urethra, may be useful. The study aimed to compare the effects of these three treatments.

**Methods:**

Subjects included patients who received TVT, TOT, or laser therapy (*n* = 50 each). The 1-h pad test, International Consultation on Incontinence Questionnaire Short Form (ICIQ-SF), and overactive bladder symptom score were used to assess the patients before and 12 months after treatment. For laser therapy, a probe was inserted into the vagina after applying a local anesthetic to the vaginal wall, and irradiation was performed for 20 min at a wavelength of 2940 nm. This treatment was performed three times every alternate month.

**Results:**

As per the 1-h pad test and ICIQ-SF, the TVT, TOT, and laser therapy groups showed comparable improvements in SUI. For patients with MUI, some in the TVT and TOT groups showed exacerbation; however, all patients in the laser therapy group tended to improve.

**Conclusions:**

The efficacy of laser therapy for urinary incontinence was confirmed. This is the first study to report on the effect of laser therapy on urinary incontinence in Japanese women.

## Introduction

Stress urinary incontinence (SUI) is a condition, wherein increased abdominal pressure, such as that from coughing, causes urinary leakage [1]; it affects up to 49% of all women. Mixed urinary incontinence (MUI) affects up to 29% of all women [2] and is a combination of SUI and urge incontinence, in which a strong urge to urinate is accompanied by urinary leakage.

Among various surgical techniques for SUI, sling procedure is effective [1]. In recent years, this procedure has been used to support the bladder neck and urethra with a synthetic material called prolene mesh tape as a sling [3–6]. In Japan, tension-free vaginal tape (TVT) and transobturator tape (TOT) are generally used for these procedures [1]. The TVT procedure involves placing a mesh tape mid-urethra, extending from the posterior surface of the pubis to the subepithelium above the pubis. The TOT procedure involves placing the tape from the obturator foramen to the vagina (or from the vagina to the obturator foramen). These procedures have shown good treatment outcomes; however, a long-term risk of complications can occur due to the artificial nature of the tape [7].

At present, a surgical procedure that does not introduce a foreign object is desirable. Attention has thus been drawn toward non-ablative erbium-doped yttrium aluminum garnet (Er:YAG) laser therapy, which consists of transvaginal laser irradiation of the urethra [8]. This laser therapy is thought to improve blood flow to the surrounding tissue and promote tissue regeneration [9]. In studies on Caucasian women, an improvement in SUI was observed, and most subjects exhibited no adverse effects [10]. A shortcoming of the treatment is that it is effective only for a few years [8–10]. However, to our knowledge, there are no comparative studies on the TVT and TOT procedures [11]. Furthermore, there are no reports on Japanese women.

## Subjects and methods

### Subject selection and study exclusion

The subject sample included women with SUI aged 20–65 years who consulted our hospital between 2014 and 2016. The presence or the absence of MUI was not considered. The preoperative observation period was 3 months, and an internist confirmed that subjects showed no objective evidence of cardiovascular disease, according to the criteria of the American Heart Association. Subjects did not have a history of surgery for incontinence or a history of treatment with drugs for incontinence, overactive bladder, neurogenic bladder dysfunction, and estrogen therapy. Other exclusion criteria included cystocele, uterine prolapse, and rectocele, as well as neuropathy such as spinal stenosis. When urge incontinence posed greater hindrance to daily life than did SUI, drugs that were not permitted in the study were administered during the preoperative observation period, and the subject was excluded. Women who were pregnant, breastfeeding, or who wanted to become pregnant were excluded. Subjects were permitted to withdraw from the study at any time.

### Evaluation method

Before and 12 months after treatment, subjects underwent a 1-h pad test [12], International Consultation on Incontinence Questionnaire Short Form (ICIQ-SF), and overactive bladder symptom score (OABSS) [1]. There are no global standards for MUI; therefore, OABSS was used, which is generally used as an indicator of urinary urgency and urge incontinence. The questionnaire consisted of question items on frequent urination in Q1, nocturia in Q2, urinary urgency in Q3, and urge incontinence in Q4. The score for Q4 was considered important for the evaluation of MUI. In Q4, patients scored the grade of MUI on a scale of 0–5 points, with 0 points: no incontinence, 1 and 2 points: mild incontinence, 3 and 4 points: moderate incontinence, and 5 points: severe incontinence.

### Surgical procedure, group assignment, and statistical analysis

The sample included 50 patients who underwent the TVT procedure in 2014 (TVT group), 50 who underwent the TOT procedure in 2015 (TOT group), and 50 who underwent the Er:YAG laser therapy in 2016 (laser therapy group), by numerical order of their medical charts. The TVT procedure was performed under lumbar anesthesia using the Advantage Fit™ Transvaginal Mid-Urethral Sling System (Boston Scientific, Co) or GYNECARE TVT™ Retropubic System (Ethicon, Inc.). The TOT procedure was performed under lumbar anesthesia using the Monarc transobturator sling system or the Obtryx II Transobturator Mid-Urethral Sling System [13].

For the Er:YAG laser therapy (FotonaSmooth™ XS, Fotona doo. Slovenia), 9% xylocaine was sprayed into the vagina. The laser probe was inserted into the vagina, wavelength was set to 2940 nm, and irradiation was performed for 20 min in the “Smooth-mode”. Irradiation was first performed for 10 min on the entire anterior wall of the vagina, then 5 min for the entire vagina, and 5 min around the urethra. In the event of cystitis, vaginitis, or menstruation on the day of treatment making the state of the vagina unsuitable, treatment was postponed until a different day. Laser irradiation was performed three times every alternate month.

All procedures were performed by the same surgeon. Student’s *t* test was used to find statistically significant differences.

### Ethical soundness

The present study was a prospective intervention study performed with the informed consent of all patients in accordance with the Declaration of Helsinki. Approval was obtained from the local ethics committee of the Lidre Medical Center (Yokosuka, Kanagawa Prefecture). There are no conflicts of interest to declare with regard to this study.

## Results

### Study subjects

For all the three groups, 50 surgeries were performed in each group, with an observation period of 12 months. The age bracket of the population in the three groups was as follows: TVT group, 48.7 ± 13.9 years; TOT group, 47.8 ± 13.9 years; and laser therapy group, 50.3 ± 13.2 years. There was no significant difference between the three groups in the pretreatment 1-h pad test and ICIQ-SF scores. For OABSS, the insertion of an artificial object was irreversible in the TVT and TOT procedures; therefore, patients with intense urinary urgency declined surgery and were started on overactive bladder pharmacotherapy first, which caused variations in OABSS scores between the three groups.

### One-hour pad test

**Fig. 1:**
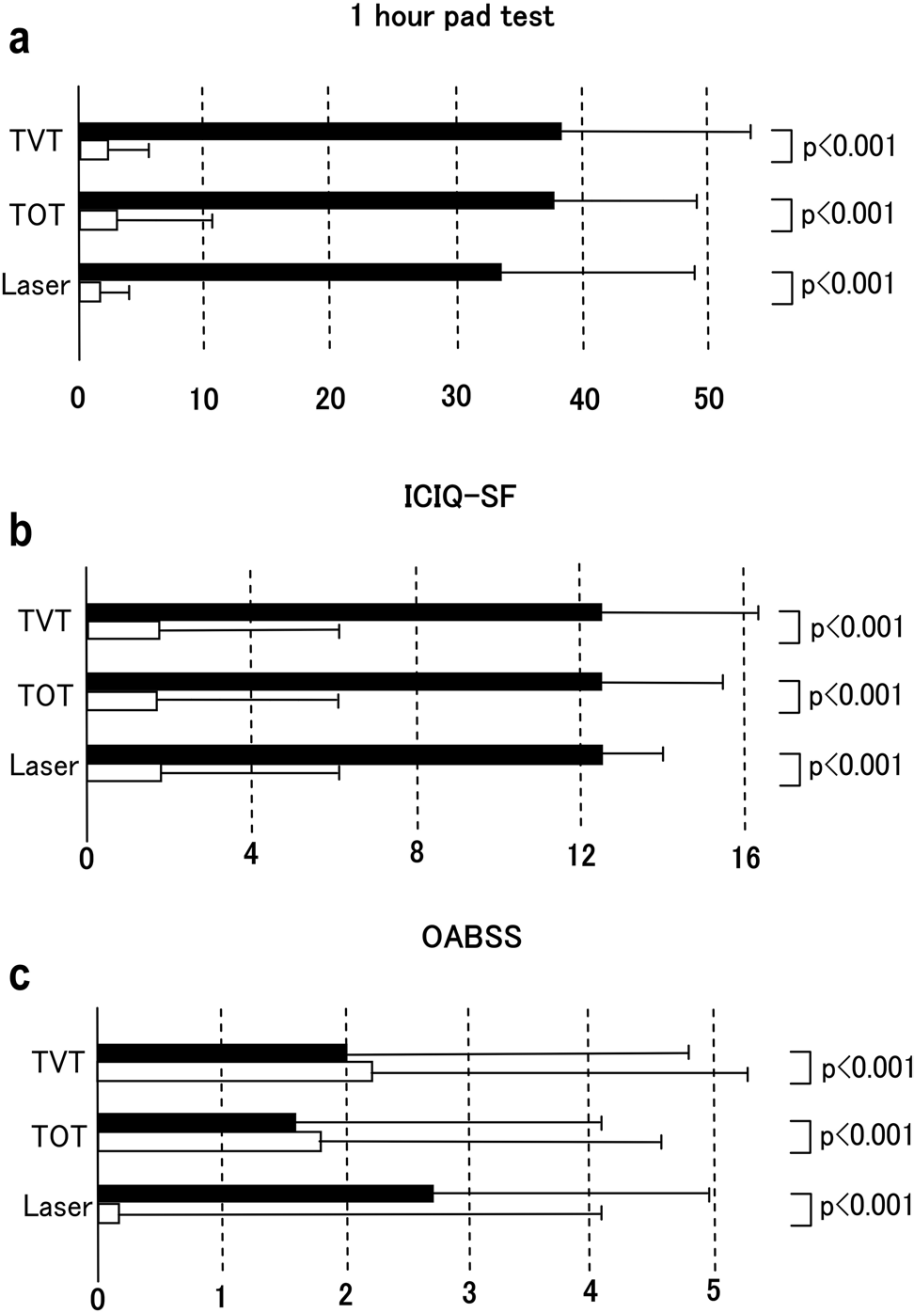
**a** Results of the 1-h pad test 12 months after treatment for the TVT, TOT, and laser therapy groups. TVT, TOT, and laser on vertical axis represent the corresponding groups. The horizontal axis indicates the quantity of incontinence (g) ± SD. **b** Results of the ICIQ-SF 12 months after treatment for the three groups. TVT, TOT, and laser on vertical axis represent the corresponding groups. The horizontal axis indicates the scores ± SD. **c** Results of the OABSS 12 months after treatment for the three groups. TVT, TOT, and laser on vertical axis represent the corresponding groups. The horizontal axis indicates score ± SD

As an objective indicator of urinary incontinence (Fig. 1a), the mean values of the 1-h pad test significantly improved in all the three groups (*p* < 0.001) after treatment. The percentage of patients with urine loss of 0 g in the TVT, TOT, and laser therapy groups were 69, 68, and 50%, respectively. Postoperative incontinence of ≥ 10 g was observed in four patients in both the TVT and TOT groups, and one patient in the laser therapy group. Among patients with urine loss of 0 g in the TVT and TOT groups, residual urine was present in only 10 and 7 patients with the mean volumes 10.1 mL and 10.4 mL, respectively. In the laser therapy group, no residual urine was observed.

### ICIQ-SF

As a subjective indicator of patient satisfaction, the ICIQ-SF score (Fig. 1b) significantly improved in all the three groups (*p* < 0.001) after treatment. Great dissatisfaction (ICIQ-SF) was expressed by three patients each in the TVT and TOT groups and by no patient in the laser therapy group.

### OABSS

Following Japanese clinical guidelines [1], we performed TVT and TOT procedures for patients with MUI. As an indicator of overactive bladder function for MUI patients, OABSS (Fig. 1c) showed great variation among the population, and only patients in the laser therapy group showed significant improvement (*p* < 0.001) after treatment.

**Fig. 2:**
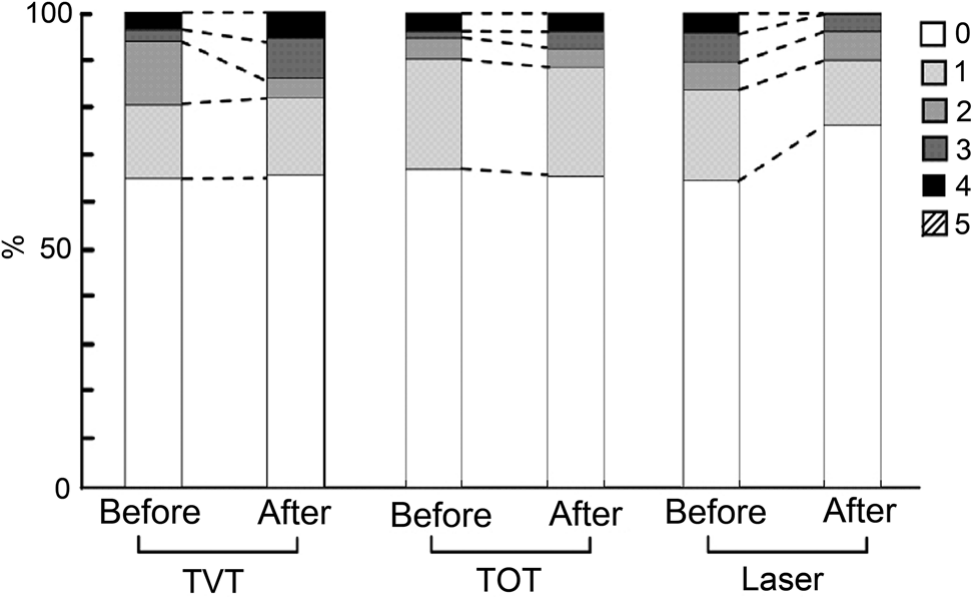
OABSS results of Q4 before and after 12 months of treatment for the TVT, TOT, and laser therapy groups. Vertical axis indicates the percentage of patients (%) grouped according to scores of 0, 1, 2, 3, 4, or 5 points. TVT, TOT, and laser on the horizontal axis represent the corresponding groups

Figure 2 shows the percentage of patients grouped according to the results of Q4 before and after 12 months. There are no patients with a score of 5 points for Q4. A pretreatment score of 4 points for Q4 was obtained in 2 and 0 patients in the TVT and TOT groups, respectively; however, the corresponding results increased to three and two patients after treatment. In the laser therapy group, 4 points for Q4 was scored by 2 patients before treatment and 0 patients after treatment.

### Complications

Short-term complications in the TVT group included severe pain in one patient and infection in two patients. In the TOT group, there was severe femoral pain in one patient and infection in one patient; however, in the laser therapy group, no adverse effects were observed. Long-term adverse effects after 12 months included persistent femoral pain in one patient in the TOT group.

## Discussion

### Findings from the present study

In the present study, the state of laser therapy has been clarified by comparing it with that of the TVT and TOT procedures. In the 1-h pad test and ICIQ-SF, comparable therapeutic results were seen for the three procedures; however, the results of the OABSS and complications differed. In other words, from the perspective of SUI, the results were comparable among the three groups; however, from the perspective of MUI and complications, laser therapy is superior. Next, the results of the present study are examined with a discussion of the literature.

### Complications and urinary urgency in the TVT and TOT procedures

Serious short-term complications have been reported in 17.3% of the TVT procedures [14], and postoperative urinary urgency occurs in 9.4% at 5 years and in 11.4% at 7 years after treatment [15]. In the present study, short-term complications occurred in 6% of patients and long-term complications occurred in 0% of patients in the TVT group; thus, the surgical techniques are considered comparable.

In the TOT procedure, short-term complications include bladder perforation in 0.4% [16] and femoral pain in 12% of patients [17]. In the present study, the incidence of complications is 4% in the short term and 2% in the long term, which shows that the surgical techniques are comparable. Due care is important, because both groups have some residual urine.

Patients with moderate preoperative urge incontinence correspond to patients with 4 points for OABSS Q4 in the present study. A postoperative increase in urge incontinence leads to postoperative exacerbation of OABSS overall and an increase in the number of patients with great dissatisfaction on ICIQ-SF. This result shows that surgery carries the risk of MUI. In the abovementioned literature, sling procedure for MUI cures urinary urgency and urges incontinence 30–85% of the times [18]. In other words, SUI did not improve, but was exacerbated in some patients. Therefore, the TVT and TOT procedures are not necessarily recommended for all patients.

In the United Kingdom, it has been reported that the use of an artificial object in female urological surgery causes problems [7]. This predicts that artificial objects may no longer be approved for use in surgery for incontinence in the UK in the future.

### Literature review of laser therapy

Laser therapy, which is addressed in the present study, is a promising new treatment. The first report [9] indicates that laser therapy may be used for vaginal relaxation, SUI, pelvic organ prolapse, and vaginal atrophy, with no serious adverse effects. The only shortcoming is that the effects of treatment are temporary and presumed to last for a limited number of years. Furthermore, there are no reports of long-term follow-up. In a cohort study [19] surveying 73 women and a prospective study [20] of 42 women, no adverse effects were observed, and the ICIQ-SF scores improved. In many reports with evaluations including the ICIQ-SF and pad test only, improvement was observed with no adverse effects. Such reports are consistent with the present study.

For MUI, one report used the King’s Health Questionnaire [10], in which an improvement was observed. However, the observation period was only 6 months. In the present study, after 12 months of observation, a comparable improvement in MUI was observed.

The mechanism by which MUI is treated includes tissue changes. It has been reported that laser therapy increased vascularization of the vaginal mucosa by 64%, and regeneration of vaginal tissue is pathologically observed in 61% [21]. It has also been reported that laser therapy improves urethral sphincter dysfunction with urethral sphincter failure [22], implying tissue regeneration.

### Outlook for laser therapy

Based on the present study and literature review, the TVT and TOT procedures are comparable for SUI; however, laser therapy is superior in terms of MUI and complications. This is an important finding, as there are no previous comparative studies of laser therapy.

According to the literature, a shortcoming of laser therapy is that it is effective for only a few years. From a positive point of view, laser therapy is considered safe, because even if unexpected adverse effects occur, they are reversible. It may be beneficial for women who desire temporary treatment and who do not want an artificial object.

## Conclusion

For SUI, Er:YAG laser therapy in women improved urinary incontinence as effectively as the TVT and TOT procedures with few complications and may be a good treatment option for MUI as well.
